# Predicting Fraud Victimization Using Classical Machine Learning

**DOI:** 10.3390/e23030300

**Published:** 2021-03-03

**Authors:** Mark Lokanan, Susan Liu

**Affiliations:** Faculty of Management, Royal Roads University, Victoria, BC V9B 5Y2, Canada; vivid_susan@outlook.com

**Keywords:** investment fraud, consumers, victims, self-regulation, machine learning, fraud prediction

## Abstract

Protecting financial consumers from investment fraud has been a recurring problem in Canada. The purpose of this paper is to predict the demographic characteristics of investors who are likely to be victims of investment fraud. Data for this paper came from the Investment Industry Regulatory Organization of Canada’s (IIROC) database between January of 2009 and December of 2019. In total, 4575 investors were coded as victims of investment fraud. The study employed a machine-learning algorithm to predict the probability of fraud victimization. The machine learning model deployed in this paper predicted the typical demographic profile of fraud victims as investors who classify as female, have poor financial knowledge, know the advisor from the past, and are retired. Investors who are characterized as having limited financial literacy but a long-time relationship with their advisor have reduced probabilities of being victimized. However, male investors with low or moderate-level investment knowledge were more likely to be preyed upon by their investment advisors. While not statistically significant, older adults, in general, are at greater risk of being victimized. The findings from this paper can be used by Canadian self-regulatory organizations and securities commissions to inform their investors’ protection mandates.

## 1. Introduction

In 2019, a report from the accounting firm MNP noted that Canadians are at risk of financial exploitation by their investment advisors [1]. (MNP, previously known as Meyers Norris Penny, is one of the largest full-service chartered accountancy and consulting firms in Canada.) In a more recent report, the Canadian Securities Administrators [2] noted that investors with specific characteristics are at higher risk of falling prey to financial exploitation than others. The individuals identified as vulnerable are not entirely comparable to stereotypical victims of financial exploitation in Canada. The Canadian Government [3] illustrated that some age groups, such as “retirees or near-retirees” over 60 years old, were more likely to fall victim to fraud than others. In contrast, other studies have portrayed the more vulnerable as “male. “middle-aged” [4]. knowledgeable with finance” [5,6,7], and “self-reliant” [8,9]. The growing number of victims, as well as the monetary loss, could tarnish the reputation of the Canadian investment industry. Protecting people who are potential targets for financial crime is not only an important public interest issue; it is also a concern for regulators to implement measures that will enhance investor protection.

Thus, it is vital to identify victim demographics, which can be used by regulators and dealers to mitigate financial exploitation. This study is aimed at predicting identifiers of financial exploitation in Canada. Using data from the Investment Industry Regulatory Organizations of Canada (IIROC), the author built a machine learning logistic regression model to predict the demographics of victims of financial exploitation. (IIROC is one of Canada’s national self-regulatory organizations responsible for policing dealer members and their registered representatives who trade in Canada’s marketplaces.) The researcher will address the following questions:

1. How do demographics affect the probability of fraud victimization?
2. Which variables are the strongest predictors of fraud victimization?

### Contribution

The main contributions of this paper are as flows:

- From a risk mitigation perspective, knowing the predictive accuracy of the probability of financial exploitation will be valuable for identifying the key factors that determine the likelihood of financial exploitation and for protecting those people from unscrupulous investment advisors.
- From a practical perspective, the predictive model shows the probability of the investors more susceptible to fall victims of financial fraud—female investors, investors with poor financial knowledge, retirees, and those investors who know their advisors from previous relationships.
- From a policy making perspective, it is hard not to see the results of this finding being used by both the national and provincial securities regulators to inform Canada’s securities regulatory framework. Canada does not have a national securities regulator and relies on the self-regulation to protect investors and sanction bad actors. It is in this regard that the proposed study is contextualized to show why a wider audience might be interested in a paper on the effectiveness of self-regulation in Canada’s securities industry and highlights the need for self-regulatory reforms and a national securities regulator in Canada.

The rest of the paper is organized into six sections. Section two reviews the literature on self-regulation and its antecedent effects from the victims’ perspective. Section three highlights the key findings from previous research on lifestyle exposure theory. Section four describes the research methodology and design. Section five discusses the results and analysis, followed by section six, which highlights the study’s limitations and areas for future research.

## 2. Self-Regulation: The Victim’s Perspective

This study focused on investors victimized by perpetrators employed by the IIROC-regulated financial institutions. Federal and provincial governments have studied and identified the personality traits of victims of financial crime at a general level, such as pre-retirees or retirees [3,10,11]. When considering demographic factors of financial victimization, the IIROC study could enable the gaining of insight from the analysis of victim demographic profiles, which would help self-regulatory organization (SRO) members intervene earlier.

The aim of this research was to track significant benchmarks seen in investor demographics, including gender, age, investment knowledge, risk tolerance, relationship to the offender and incidence of financial crime. By examining investor demographic profiles, the author could detail factors that previous researchers have overlooked.

This study comes at an opportune time when securities regulation and investor protection is at a crossroads in Canada. The country has a myriad of regulators controlling its marketplace. The CSA is the umbrella organization responsible for overseeing provincial and territorial securities regulators. whose objective is to improve, coordinate and harmonize regulation of the Canadian capital markets” [2] (p. 1). Within this mix, SROs have the power to regulate the securities and mutual funds dealers under the supervision of provincial commissions. The primary SROs in Canada are IIROC, the Chambre de la Sécurité Financière and the Mutual Fund Dealers Association of Canada (MFDA). The SROs can impose administrative sanctions to their members, including fines, suspension and license revocation. In cases where the infractions have criminal elements, the matter is referred to the courts. Canadian SROs are nongovernmental organizations that play “a compensatory role” [12] (p. 405) for provincial regulators [8,13]. For decades, SROs have played a vital role in regulating Canada’s investment industry and promoting investor protection by purporting to regulate in the public interest [14]. Recent developments have seen some provincial regulators delegating “additional statutory investigative and fine-collection powers” to these SROs [14] (p. 3).

Despite these layers of safeguards, there are serious concerns about investor protection and vulnerability to financial exploitation. One of the reasons for these concerns is the inherent conflict of interest between the SROs’ efforts to balance the industry they govern and the protection of the public interest [15,16,17]. The CSA’s consultation paper pointed out that “[c]lients are not getting outcomes that the regulatory system is designed to give them” [17] (p. 8) and that effectively, there is a need for a frontline regulator that will enhance investor protection and Canada’s capital markets. In June 2020, the CSA published a consultation paper requesting feedback on whether the current SRO framework “best serves Canadian investors and the investment industry, in light of the evolution of the financial services industry” [2] (p. 1). The CSA is asking for comments on SRO frameworks that will enhance capital market governance and protect investors from financial exploitation. This paper was written to inform the CSA, provincial regulators, SROs and industry participants about the demographics of investors who are vulnerable to financial exploitation.

A second contribution of this paper concerns the proposed MFDA and IIROC merger. In December 2019, the CSA announced that it will conduct a review of these two dominant SROs that are part of Canada’s regulatory framework to better understand their investor protection mandates. Louis Morisset, CSA Chair and President/CEO of the Autorité des Marchés Financiers (AMF) (AMF is responsible for financial regulation in Quebec) said:

The regulatory framework for these self-regulatory organizations has been in place for several years, and the industry has evolved significantly during this time. In response to requests formulated by market participants, we believe it is appropriate to revisit the current structure and seek comment from stakeholders. [18] (p. 2).

The MFDA is envisioning an approach that will merge all SROs into a single agency covering all dealers and their members, while leaving market regulation to the CSA [16] (p. 1). The proposal would have a variety of benefits for Canadians, including enhancing confidence in securities regulation and protecting investors from financial exploitation [16] (p. 9). Ermanno Pascutto, Executive Director of the Canadian Foundation for Advancement of Investor Rights [19] (p. 10) said:

“*Merely merging the two SROs using the current self-regulatory model would not be adequate given the shortcomings of the current SRO system. The important question is would a consolidation be in the best interests of the investing public and in the public interest? We urge the CSA to consider a new self-regulator model and SRO organization.*”

Pascutto went on to assert that “the nature and meaning of the SROs’ public interest responsibility, and how the CSA can ensure that it is met, should be carefully considered” [19] (p. 10). It is expected that this paper will inform the response to the Ontario Securities Commission’s taskforce on securities modernization and the call for the unification of the MFDA and IIROC into one SRO. It is important to note here that the taskforce is not calling for the MFDA to be subsumed by IIROC but rather for the creation of a new, single SRO with an enhanced accountability and governance structure, where the functions of the MFDA and the IIROC will be rolled into one (and later expanded to other registration categories). The argument is that the creation of a new SRO will empower CSA statutory regulators and restore public confidence in the SRO model, whereas a simple merger of IIROC and the MFDA would merely perpetuate the status quo. This paper will inform those assertions and provide valuable insights for the taskforce to consider.

## 3. Theory and Literature Review

### 3.1. Lifestyle Exposure Theory

This paper is anchored in lifestyle exposure theory (LET). Although LET was developed as a theoretical model to study street crime, it also offers a promising conceptual framework with which to study victimization by financial fraud [5]. The central tenet of LET is that victims are perceived as individuals who expose themselves to risky situations [20]. Lifestyle is defined as “routine daily activities, both vocational activities (work, school, keeping house, etc.) and leisure activities” [20] (p. 241). Accordingly, the more that individuals are in situations or environments that expose them to potential offenders, the more likely it is that they will experience victimization [21] (p. 167). Victimization then is based on the premise that different lifestyles lead to differential exposure to criminogenic environments in which victimization is likely to occur [22] (p. 901). To go further. different lifestyles are associated with differential risks of being in particular places, at particular times, under particular circumstances, and interacting with particular kinds of persons” [23] (p. 469). It is not simply being in a criminogenic environment and dealing with high-risk individuals that matters but also the differential risks associated with being in an environment and the individuals that influence one’s susceptibility to victimization [24] (p. 104). LET involves considering how lifestyle affects the risk or odds of being victimized and therefore can be seen as a probabilistic theory [22,25].

The idea of victim facilitation in crime can be extended to financial exploitation [21]. In applying LET to victim exposure, it is important not only to examine how deviant lifestyles influence victimization [26] but also to understand the level of adaptation and cooperation between victim and offender where the latter sees the former as prey [27]. Pratt et al. [24] suggested that when it comes to financial crime, the behaviors that are considered risky are different from those considered risky in street crime contexts. In financial crime victimization, there is more emphasis on spatial convergence and network, where vulnerable individuals are exposed to predatory offenders [28]. It is not time spent away from home that is considered risky, but time spent in criminogenic culture (i.e., culture conducive to fraudulent conduct) where victims are exposed to predatory individuals who try to gain their trust and exploit them for their savings [27].

Within this mode of normalization, the dynamics of facilitating financial exploitation are different from the dynamics observed between offender and victim in traditional street crime. In the case of financial exploitation, the “interaction between offender and victim is cooperative … victimization requires that the victim participate or go along with the offender to some degree” [21] (p. 167). The perpetrator(s) knows the victim will not approve of his actions if he is detected, so he creates rationalizations that minimize his exploitative actions [4]. From a psychodynamic viewpoint, the internalization of criminogenic behavior represents a profound alienation from the larger group to which the perpetrator(s) belongs [27]. By virtue of their positions as experts, perpetrators learn to see themselves as more acted upon than acting; they prepare the way for deviance from the dominant normative system without the necessity of a frontal assault on the norms themselves [29] (p. 667).

Most of the previous research on LET has been conducted on street-level crimes [22,23,24,26]. Lifestyle research in this area tends to be focused on victims’ association with offender and exposure to criminogenic environments as strong predictors of victimization [22,24]. However, with the increase in financial crime, researchers have begun to explore the empirical utility of applying LET to financial victimization [24,28,30]. Among the studies to date, there is no consensus on the characteristics that make individuals more vulnerable to victimization. Research suggests that the “demographic profiles of fraud victims are difficult to observe” [31] (p. 206), that the notion of a “typical fraud victim” does not exist [28,32,33]. Instead, the findings that have emerged from fraud victimization literature have singled out various demographic traits as being more vulnerable to exploitation than others.

There are mixed reviews between gender and fraud victimization. Holtfreter et al. [31,32] found evidence to suggest that males are more vulnerable to fraud victimization than females. Lokanan [4] also found that males are more likely to be victims of fraud than females and attributed this to aggressive investment strategies. In general, however, there does not seem to be a consensus on the relationship between gender and fraud victimization [28,31,34,35,36]. When looking at the effect of other demographics, namely knowledge, income and occupation, on fraud victimization, the results are inconsistent across studies [4,28,31,34]. That said, the findings do provide important insight into demographics that may increase the risk of fraud victimization.

Age is one of the demographics frequently associated with fraud victimization [4,28,34,36]. Older adults are more likely to be victims of fraud than younger adults [28,32]. Lokanan [4] supposed this was related to older clients siting on large cash reserves they want to invest to secure retirement. However, other studies have shown that younger adults are victimized because they take more risks and are more aggressive in their investment decisions [5,33,36]. The one commonality between groups is that, unlike predatory offenders in street-level crimes, financial fraudsters are typically relatives and long-time friends who gain the trust of their victims [4,5,37]. These people have been exposed to the fraudsters through day-to-day interactions and via legitimate means, which has created opportunities for them to be victimized.

Synonymous with age, retirees are another group that is susceptible to fraud [4,5,28]. A series of studies have shown that retirees may disproportionately be targets and victims of fraud [28,38,39]. One reason for this is that retirees more frequently want to ensure they have enough funds set aside for retirement and hence are more prone to exposure to fraudsters [4,38,39]. Collectively, these increased investments create opportunities for financial exploiters to take advantage of their existing trusted relationships with elders and their social networks to seek out potential targets [5,39].

### 3.2. Fraud Victimization

Financial crime erodes public trust and affects the stability of the markets [40] (p. 5). The behavior and action that constitutes financial crime has always been difficult to define [41,42,43]. The uncertainty in the definition of financial crime raises questions about the demographic profile of investors who are more vulnerable to financial exploitation. Generally speaking, financial crimes are broadly defined as nonviolent acts committed by someone in a position of authority that violate the criminal code and result in financial loss [40,41,44,45,46].

Financial exploitation is becoming a serious problem in many countries [45,47]. Financial exploitation scenarios vary but can include forging signatures, misusing assets, gaining unauthorized access to accounts and permitting discretionary trade [5,11,13,27,45,48]. Much of the literature on financial exploitation highlights that its purpose is to maximize financial gains [3,5,45,49]. Given that financial exploiters predominantly target seniors [5,11,13,47,48], greater attention has been given to policies designed to prevent victimization among this demographic [4,50,51]. Researchers in this area have focused on why seniors are more vulnerable to financial exploitation than other age groups and found that the health-related effects associated with ageing, such as cognitive impairment, affect decision-making [3,5,50,52,53]. Other scholars have found that most financial exploitation is committed by advisors in a position of trust, such as family members, care providers or longtime close friends [3,4,5,7,10,45,50,53]. Unlike fraudsters who tend to ensnare their targets using professional tactics [5,45,54,55], financial exploiters have already earned the trust of their victims, enabling them to invade their accounts and cause irreparable economic and financial harm [4,5,50].

The financial crimes related to the 2008 global financial crisis triggered a public outcry that resulted in a large body of scholarly work examining how financial literacy levels are associated with exploitation, as well as traits of victims of these crimes. Researchers in this area tended to focus on older adults and found corroborating evidence to show that seniors are more vulnerable to financial crime than any other age group [3,4,7,48]. Frequently cited scholars noted an interactive effect among the elderly, poor investment knowledge and financial exploitation [3,4]. Others found that seniors who are victims of financial fraud suffer from health-related problems (mainly cognitive decline) and are unable to make sound investment decisions [3,4,5,53]. There is empirical support for this view in the existing literature.

In the recent past, researchers have focused on the association between a victim’s age-related problems and financial victimization, especially when mental health issues are involved [5,7,53]. A review of the literature on the effect of mental illness showed that cognitive impairment is strongly associated with behavioural changes and may contribute to victims falling prey to financial crime [5,48,50,52,53,56]. Other cognitive impairments are instrumental in the increasing likelihood of vulnerability to financial crime [7,57]. One such factor is “doubt deficit” [50] (p. 3). Doubt deficit is linked to health-related problems that have led seniors to rely more on financial professionals to make investment decisions [3,5,50,53]. More specifically, doubt deficit caused by cognitive changes affects the financial decision-making of seniors and consequently increases the probability of victimization [5,48,50,52,53,56].

### 3.3. The Present Study

The current study builds on the existing body of literature on fraud victimization and the LET framework to predict the demographics of those vulnerable to fraud. In employing LET as a theoretical framework, an important question for financial market regulation scholars to consider is whether victims put themselves at risk by engaging in risky investments or whether the victimization is a by-product of their trusting relationship with their investment experts. Although different variations of LET have been applied to the study of financial exploitation and fraud, to date no researchers have examined the differential risk of victimization by investment fraud. This study is an attempt to fill this gap by utilizing LET to build a machine learning algorithm for predicting the demographic traits that are at risk for fraud targeting and victimization.

## 4. Research Design

### 4.1. Financial Victimization Detection Model

Existing research has been focused on the traditional statistical techniques used to identify factors that increase the probability of fraud victimization [5,58]. Other scholars have assessed the likelihood of vulnerability to different types of financial crime by applying computational and statistical methods [12,59]. The two most common techniques are neural networks [49,60] and binary logistic regression, respectively [41,50,61,62]. Neural networks could effectively be employed to identify patterns in large unstructured datasets [12,58,59]. On the other hand, logistic regression analysis can be used to build classifiers and predict an outcome [12,58,59]. Additionally, logistic regression requires that the observations are not interrelated with one another [12,59]. However, predicting classification outcomes has become easier with scikit-learning models in Python programing language, which can be used to analyze unbalanced datasets. This is particularly useful in this study because the number of people who are victimized is proportionally less than those who are not. Using Python, a machine learning logistic regression model can be built to make predictions based on fraud victimization data. More specifically, a machine learning predictive model can be employed to identify the demographic factors that increase the probability of falling prey to investment fraud. 

### 4.2. Data Collection

The author collected data for the study from the enforcement cases heard by IIROC District counsel enforcement panels. The IIROC enforcement system is a platform that lists the decisions and reasons in cases after a hearing. The information collected from the cases included the type of financial crime and offender and victim profiles. The researcher gathered data on victims’ variables through two rounds of coding to provide insight into the demographic profile of investors. 

### 4.3. Data Coding

The first round of coding involved identifying the victim-related cases. The author retrieved a total of 616 cases from 2006–2019 from the IIROC enforcement database. The initial step in the coding process was to peruse a sample of cases to identify victim demographic variables for further empirical analysis. Once the researcher had identified the variables, she coded a sample of 10 cases between two coders to verify both accuracy and reliability. The second round of coding involved sorting the cases that dealt with victims of investment fraud. This led to a total of 235 cases. From these cases, the scholar identified a total of 4575 investors as victims. It is important to point out that there was more than one victim per case, for a total of 4575 victims. Owing to missing values for some variables, the dataset used consisted of 383 victims from 187 cases. 

### 4.4. Description of Variables

#### 4.4.1. Predictors Considered

Prior researchers found evidence to suggest that gender, age, occupation and education level are associated with fraud victimization and financial risk [10,57,63]. To some extent, financial risk-taking was found to be associated with greater risk of victimization [27]. Following the lead of the fraud victimization literature on the impact of lifestyles-related outcomes, the author used the following variables to predict targeting for fraud victimization.

#### 4.4.2. Target Suitability

- Gender

Gender of victims is a vital measure in the study of victimization and financial criminality [4,64,65]. Previous researchers have tested the gender difference in victimization as one of the control variables, while using other variables to identify the predictive effect on fraud victimization. In this study, the gender difference (male or female) is dichotomized with null coded as 0 and vice versa.

- Age

Age has been used in regression models to predict vulnerability to fraud [7,37,39]. A common belief is that seniors (age 65 and above) are more likely to experience fraud victimization in Canada [4,10,56,66]. In this study, age is measured as a continuous variable in the logistic regression model.

- Occupation

Occupation status is a variable used in previous research to provide insight into the demographics of fraud victims [4,67]. In this study, occupation is coded as a categorical variable classified into three categories to align with employment status in Canada. The author converted each value of this variable into a dichotomous variable as either non-null value (coded 1) or no value (coded 0). Occupation was coded as employed = 1 or 0, unemployed = 1 or 0 and retired = 1 or 0.

- Investment knowledge

Financial literacy is another measure often used in the literature to provide insight into fraud victimization [4,61,67]. The research in this area provides conflicting results. On one hand there is evidence showing that a lack of financial literacy is an indicator of fraud victimization [3,4]. However, more recent research has indicated that the number of fraud victims who are financially literate is proportionately higher than those with limited financial knowledge [9,57,68]. In this study, the researcher coded investment knowledge as a categorical variable classified it at three levels: poor, moderate and good. Each value of the variable was dichotomized into non-null value (coded 1) or null value (coded 0).

- Financial loss

Financial loss is a continuous variable that provides insight into the magnitude of the economic harm to the victim. Prior researchers found that investment fraud victims are more likely to sustain heavy financial losses than victims who suffered from other types of financial crimes [4,67]. In a study of elderly fraud, DiLiema [5] found that the loss was around $200,000 or above per victim. While studying investment fraud in Canada, Lokanan [4] found that 41% of victims lost $25,000 or less and 23% lost $100,000–$200,000.

- Offender–victim relationship

Relationship to the fraudster is a variable often used in fraud victimization studies to measure trustworthiness [4,67]. Previous research indicates that a trusting relationship is strongly related to financial victimization [4,5,10,13,45,54]. The offender–victim relationship variable was coded as a categorical variable and classified as family member, friend, acquaintance, known from the past or other relationship (e.g., introduced by a third party). Each indicator is coded 1 for non-null values and 0 for null values.

#### 4.4.3. Target Variable

The dependent variable is fraud victimization. Fraud victimization is conceptualized as individuals who suffered economic harm as the result of their investment advisors’ action. Fraud victimization is a dichotomized variable and coded 1 for committed and 0 for did not commit.

#### 4.4.4. Model Strategy

All of the analysis was performed using Python programing language in Scikit-learn. Scikit-learn is a very popular machine learning library for Python and was used to build the algorithms. The evaluation of classification algorithms was done using confusion matrix, while their performance was analyzed using the following measures: accuracy, sensitivity, specificity, precisin, F-measure, and area under the curve (AUC). The prediction capability was evaluated using K-fold cross validation to pick the algorithm with the highest predictive accuracy.

#### 4.4.5. Preprocessing

In the preprocessing stage, missing data were identified and imputed using mean, median, and mode. Mean imputation was used when there were normal distributions of the numerical features. On the other hand, the median was used to impute for the missing values when the features were right-skewed. The categorical features (gender, occupation, investment knowledge, and victim-offender relationship) were transformed into dummy variables. The mode was used to impute the missing values from the categorical variables. The data was split using an 80/20 train/test hold-out validation. The models was then trained on the training set and evaluated on the test set to see how they perform on unseen data (i.e., generalization measures). The standard scaler normalization technique was used to rescale the numerical values so that they ranged from 0 to 1. To check for multicollinearity, a pairwise correlation that accounted for higher than 0.70 was check among the predictors. The Recursive Feature Elimination (RFE) technique was employed to rank the most important predictors for the models (65). Cramér’s V Pearson Chi-Square coefficient matrix was use to test for multicollinearity among the categorical variables. The cut off was set at a *p*-value of 0.70 (77).

#### 4.4.6. Class Imbalance with SMOTE

In this study, data were cleaned and explored using Python 3.7.6. While analyzing the pooled IIROC dataset, a ratio analysis indicated that the fraud sample (i.e., the target variable) was imbalanced. Out of 383 cases, 289 (75%) were related to fraud victimization and 94 (25%) were not related to fraud victimization, for a 3:1 imbalance ratio. The risk of using imbalanced data in logistic regression with Python is that either the train or the test model could end up containing all or a significant portion of the fraud victimization cases. In this regard, the learning model places more emphasis on the majority class that occurs more frequently [45,55]. Consequently, the study runs the risk of underestimating the traits of the minority, which could result in a false-negative rate [45,55]. To tackle the imbalanced data issue, the oversampling or undersampling approach is an efficient means of either increasing the minority class observations or removing the majority class instances [24,55]. Because the number of observations of nonfraud victimization was 94, we employed the oversampling approach to reclassify and balance the data [24,55,69]. 

The class imbalance problem generally affects the quality and reliability of the results in machine learning tasks. Specific techniques and quality measures such as Synthetic Minority Oversampling Technique” (SMOTE) should address these issues (99,3). In this paper, SMOTE was used to over-sample and balance the target variable by adding new fraud victimization cases (up-sample) through linear interpolation (56,48,74).

More specifically, minority oversampling technique was used to synthesize new observations for the minority class (i.e., fraud). Minority oversampling is a technique that increases the number of observations by joining the *k* neighbours of the minority class nearest neighbours through synthetic samples instead of over-sampling with added observations to prevent over-fitting problems. After synthesizing new minority examples, the minority and majority classes’ sample size is equalized to form a balanced dataset. Minority oversampling creates more synthetic samples for the training set using SMOTE. In effect, minority oversampling creates new synthetic elements where the algorithms can make classification not only on the non-fraud class; but also on the fraud class.

Note also that SMOTE was only used to reclassify the imbalance of the target variable fraud victimization. Even though there were binary features in the model, they were converted into categorical features (0, 1) to use SMOTE. In this regard, SMOTE was used to up-sample and increase the number of no-fraud victimization (i.e., the minority class) cases and combine them with the majority class cases (i.e., fraud victimization). By combining the up-sampled observation from the minority class, we created a balanced dataset without modifying the majority class original observations.

#### 4.4.7. Parameter Optimization

The logistic regression algorithm uses class probabilities that are estimated by the maximum likelihood estimation (MLE). The MLE estimates the model coefficients and uses a probability distribution for the dependent variable optimized to determine the set of parameters that results using the training set. The parameters use a random state equals 0, with a test size of 0.2. The Naïve Bayes Classifier parameter is the probability of the given features over the target variable and the conditional probability for each class. The split point employed was 0.2, and the random state was 0. For the SVM model, the regularization parameter was C = 1.0, the kernel used was “linear. and the default degree equals three. The train/test split was 0.2, and gamma was set to auto. For all the algorithms, the parameters used to predict the dependent variables were yes for fraud victimization and no non-victimization.

### 4.5. Machine Learning Algorithms Considered

#### 4.5.1. Logistic Regression Algorithm

The researcher applied a logistic regression model to predict the probability of the demographic traits of people likely to be victimized. Logistic regression is a predictive technique widely employed to solve problems when there is a classification of a binary (only two possible outcomes), dichotomous, dependent variable [70,71,72,73]. Logistic regression is a good fit for this study because it provides a probability score for the observations as well as the accuracy of the prediction [70,71]. Because the dependent variable in this study (fraud victimization) has a binary outcome (coded as 0 or 1), a logistic regression model was deemed a good technique to analyze and provide insight into the findings. Informed by prior research, the author hypothesized the probabilistic effects of the demographic traits on the likelihood of fraud victimization, as follows:

$$\ln\;(\frac{P_{i}}{1-P_{i}})\;=\;\beta_{0}\;+\;\beta_{1}*\mathrm{gender}\;+\;\beta_{2}*\mathrm{age}\;+\;\beta_{3}*\mathrm{occupation}\;+\;\beta_{4}*\mathrm{investment}\;\mathrm{knowledge}\;+\;\beta_{5}*+\;\beta_{7}*\mathrm{monetary}\;\mathrm{loss}\;+\;\beta_{8}*\mathrm{offender}–\mathrm{victim}\;\mathrm{relationship}\;+\;e_{i}$$

#### 4.5.2. Naïve Bayes Classifier

Naïve Bayes Classifier (NBC) is a classification technique that is based on Bayes’ Theorem probability theory. The main assumption of NBC is that all the predictive features are independent of each other. In simple terms, NBC assumes that all the independent variables in the model are unrelated to any other features when predicting the outcome. There is no assumption of multicollinearity in the NBC model. That said, NBC is very useful when dealing with large datasets and with many features. In this sense, NBC is said to outperform other classification models. NBC provides a way to calculate the posterior probability *P*(*c*|*x*) from *P*(*c*), *P*(*x*), and *P*(*x*|*c*) and is represented by the following equation:

P(y|x) = P(x|y) × P(y)/P(x)

where:

*P*(*y*|*x*) is the posterior probability of class (Y, target) given the predictor variables (*x*)
*P*(*x*) is the prior probability of class *x*
*P*(*y*|*x*) is the likelihood given the predictor *x* of a given class
*P*(*x*) is the prior probability of predictor *x*

#### 4.5.3. Support Vector Machines (SVM)

**Figure 1:**
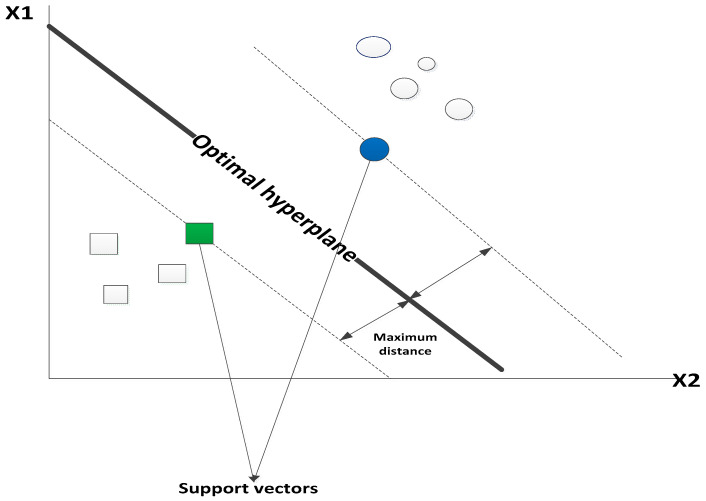
SVM Hyperplanes.

SVM is another machine learning algorithm that is used for classification problems. Unlike other algorithms, SVM uses a hyperplane, which acts as a decision boundary between the two classes. SVM was picked for this model because it contrasts with the NBC model in that it works on more complex datasets. SVM algorithms work to project nonlinear samples and look at the dataset’s extremes to draw a decision boundary known as a hyperplane (37). The primary objective of SVM is to align on a hyperplane, X number of features that classify the data points into two segments (in this case, fraud victimization and non-fraud victimization). Many hyperplanes can be selected; the objective is to find the one hyperplane with the maximum distances between the other two surrounding planes. Figure 1 below shows how this works. Only the points (support vectors) closest to the hyperplane contributes to the algorithm. The support vectors are the data points that the margin pushes up against or the points against the sample’s opposing class (37). Essentially, SVM works as a support vector, which best segregates the fraud victimization and non-fraud victimization classes (37). SVM algorithms allow for optimizing the decision boundary and classification of the target variable. Like the other algorithms, SVM learns from the training data and then decide on the unseen test data. When the decision boundary is not optimized, the resultant effect is more significant misclassifications of the data. The drawback of SVM is that they do no directly provide probability estimates. These are calculated using the expressive K-fold cross-validation. The equation for SVM is as follows:

**w**^T^**x** + 𝑏 = 0

where **w** and **x** are the weight vectors, 𝑏 is the bias.

## 5. Findings and Analysis 

### 5.1. The Vulnerable Fraud Victim

**Figure 2:**
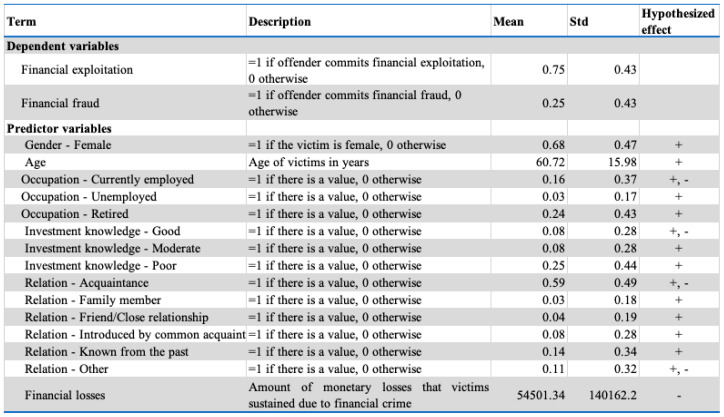
Descriptive Statistics.

Figure 2 presents the descriptive statistics for the demographics associated with fraud victimization. The age gap between the two classes was not statistically significant. The average age difference between victims and non-victims was approximately three years. The more vulnerable age group was victims aged 60 years and older. There was a statistically significant difference in the average financial status between victims and non-victims. Victims of financial fraud had more liquid household assets and net worth but less annual household income than non-victims. Note also from Figure 2 that investors who were *not* victimized suffered more significant losses than investors who were victimized. These results are similar to fraud victimization findings observed in other research [50,61].

**Figure 3:**
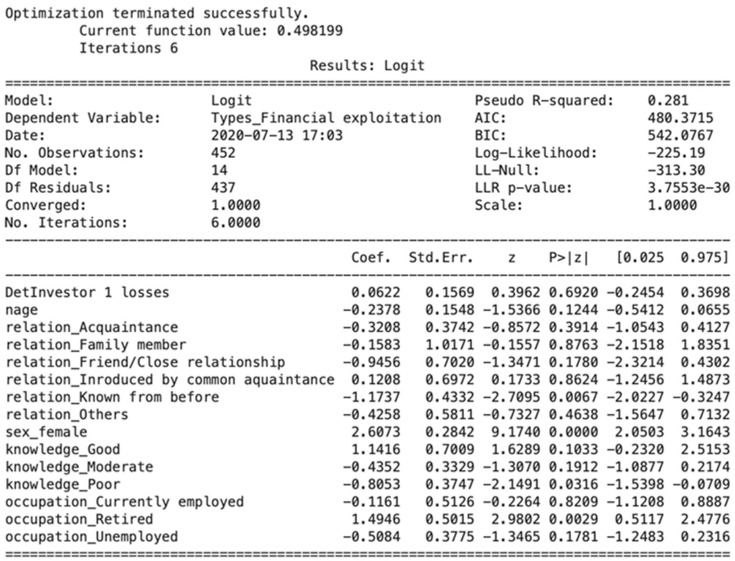
Probability of Fraud Victimization.

Results from the logistic regression model for the coefficients and odds ratio (OR) are presented in Figure 3. As seen in Figure 3, the likelihood of victimization was contingent on changes in gender, relationship with the offender and employment status. For those victimized, gender difference was one of the factors that significantly related to the victimization (*p*-values < 0.001). Accordingly, each one-unit change from male to female could increase the log odds of being a victim by 2.61 and could also increase victimization by approximately 14 times more than male. Hence, the results show that female investors are more vulnerable to financial exploitation than males. Note, these findings go against previous research, which stated the males were more likely to be victims than females [4,31,34].

The results suggest that female investors are more susceptible to fraud than males for the following reasons: First, Canadian women are primarily responsible for financial management of their households. This responsibility increases their risk of being victimized by unscrupulous advisors [74]. The Canadian Imperial Bank of Commerce [74] (p. 12) pointed out that female residents of Canada owned and managed around “$2.2 trillion of financial assets.” The value of assets Canadian women manage is expected to double in the next 8 years (Canadian Imperial Bank of Commerce as quoted in [74] (p. 13)).

Second, the unemployment rates for women have remained steady over the past 2 decades. Statistics Canada reported that the year-over-year (YOY) growth rate in female workforce participation was approximately 4% [75]. Females’ increased workforce participation has led to more stability and an upward trend in their annual income [76]. Consequently, the change in Canadian women’s financial position and independence has empowered them to become more involved in the investment decision-making process [77].

Third, evidence suggests that females have less confidence in their financial knowledge and ability to make strategic investment decisions compared to males [4,77]. As a result, females rely more on advice from professionals [77]. For instance, in a recent IIROC case, the panel summarized that the victim, TR. relied on the [r]espondent’s advice and consented to his recommendations, but did not understand them” because she “had limited investment knowledge and no experience with, or knowledge of, options trading” [63] (pp. 5–6). These limitations expose female investors to risky financial plans and increase their probability of fraud victimization.

Figure 3 also shows that the coefficient on long-term relationships between investors and financial advisors is statistically significant but negative (*p*-values < 0.05). For investors who have a long-term relationship with their advisor, the probability of being a victim is reduced by 69%. These findings contradict the empirical literature that shows that most fraud victimization results from relationships with close friends, relatives or long-term financial advisors [4,5,10,13,54]. The result seems quite natural because a long-term relationship could improve cooperation and understanding of the investor’s financial needs [4]. Every comprehensive financial plan is based on the premise that the financial advisor is completely in line with the investor’s interest and financial needs (EY as quoted in [78]). Any violation of this understanding can lead to mental and financial harm, which, could undoubtedly be a huge threat to a trusting relationship. Interestingly, in some of the cases examined, fraud occurred because the offender took over the clients’ accounts from other advisors and had never met or contacted them before doing so. In the case of IIROC vs David Hayes [79] (p. 4), the hearing panel noted the following:

“*[The offender] took over operation of both EM and PM’s RRSP accounts in early 2003 as the primary advisor. At this point, the [r]espondent had never met EM nor had he ever spoken to her*”.

In the other case of IIROC vs George Alexander Pedar Pedersson [62] (pp. 4–6), the offender tailored the unsuitable financial plan that resulted in the victim’s loss because he failed to know the victim fairly well. In their decision, the panel surmised that during the period (the victim “SW” knew the offender 2 weeks before). the [r]espondent failed to fully consider essential facts related to SW, such [as] her age, her limited future earning potential, and the total value of her retirement portfolio” (p. 6). The unfamiliar relationship between the offender and the investor could have contributed to financial victimization. 

Parallel to the investor’s relationship with the advisor, poor investment knowledge has a significant but negative effect on fraud victimization (*p*-value < 0.05). As seen in Figure 3, investors classified as having poor investment knowledge could reduce the log odds of being victimized by 0.81 and have an estimated OR of falling victim to fraud of 0.45. In other words, for investors with poor financial literacy, the probability of falling prey to financial fraud dropped 55%. These findings do not appear to be in line with the previous research on financial literacy and fraud victimization. The reasoning for this could be that, as previous researchers have found, investors who are knowledgeable about finance are “self-reliant and self-deterministic” and rely on their knowledge and experience over that of financial professionals [9] (p. 7). 

As is further evident in Figure 3, an employment status of retired has a positive influence on fraud victimization. A *p*-value of < 0.05 indicates that being retired has a strong positive correlation with fraud victimization. Each unit change in being retired can increase the OR 4.46 times. These results corroborate the findings of previous research that being retired is strongly correlated with fraud victimization. Prior researchers found that retirees were more vulnerable to being victimized for three reasons: their current financial status [50], fear of not having enough money for retirement [4,53,80,81] and social isolation [5,50].

Other scholars found that retirees are sitting on large amounts of cash, which makes them vulnerable to fraud [49,50]. Financial insecurity, coupled with the possibility of running out of money in retirement and the promise of high returns on investments, puts seniors at high risk for exploitation [4,53,80,81]. Synonymous with this argument is that seniors who are suffering from social isolation after retirement are seen as targets by motivated offenders who gain their trust because they are generally more trusting [50,82]. Socially isolated retirees are also more likely to experience physical and cognitive decline [82].

### 5.2. Accuracy of the Predictive Models

**Figure 4:**
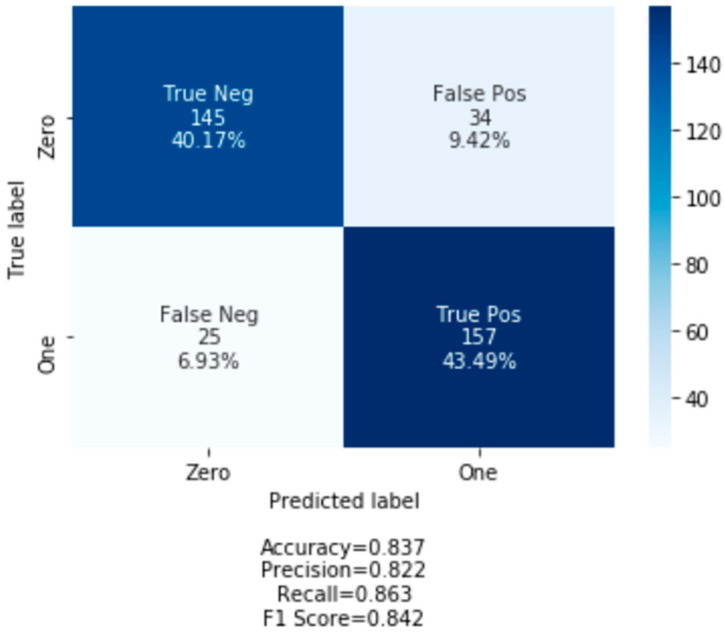
Confusion Matrix of Fraud Victimization Model.

A confusion matrix was used to further evaluate the classification model. In a confusion matrix, the number of correct and incorrect predictions is summed up based on the predicted and observed classes. Figure 4 presents a heat map of the prediction and shows that the model has an 83.66% (i.e., True Negative + True Positive) accurate prediction and a 16.34% (False Positive + False Negative) incorrect prediction of people who are likely to be victims of investment fraud. A more in-depth examination of the confusion matrix illustrates that the model predicted that 40.17% (True Negative) of the people who were predicted to be victimized were actually victimized. At the same time, the model predicted that 43.49% (True Positive) of people would not be victimized, and they were not. Only 9.42% (False Positive) of the time did the model predict that investors would be victimized when they were actually not victimized. Only 6.93% (False Negative) of the time did the model predict that investors would not be victimized when they actually were victimized. Similarly, the NBC classifier accuracy rate was 73.41% which was lower than the logistic regression accuracy rate. The accuracy rate of the SVC model was 76% and was much closer to the logistic regression model.

**Figure 5:**
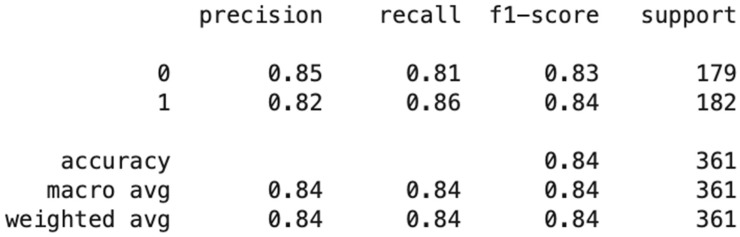
Model Accuracy and Classification.

The classification model was further evaluated from three dimensions in the confusion matrix: precision, recall and F1 score. Precision evaluated the proportion of predictions that the model predicted as actually true. As seen in Figure 4, the model is correct in predicting fraud victimization 82% of the time. The recall/sensitivity column evaluates the proportion of clients predicted to be victimized and actually were victimized. The results in Figure 5 show that the model predicts fraud victimization in the test set 86% of the time. The F-beta score is a weighted harmonic mean between precision and recall and disclosed the model’s accuracy of 84%. As seen in Figure 5, of the entire pooled IIROC dataset, these three parameters illustrated that the accuracy of the classification model was predicted to be 85%. 

**Figure 6:**
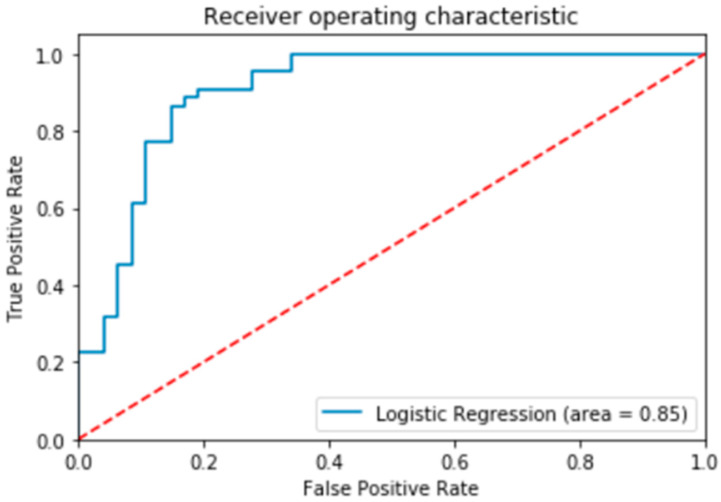
ROC Curve of Financial Exploitation.

The Receiver Operating Characteristic (ROC) curve is extensively employed to demonstrate the performance of a predictive model. The ROC is a plot of the true positive rate against the false positive rate [83]. As seen in Figure 6, the Area Under the Curve (AUC) score is 0.85. An AUC score of 1 indicates a perfect classifier, while a score of 0.5 represents a weak classifier. The predictive model has a higher discriminative capacity when the ROC curve stays as far away from the dotted line as possible [83]. An ROC score of 85% indicates that the model has a very good probability of predicting fraud victimization. 

## 6. Discussion and Conclusions

Fraud victimization is a common occurrence in the Canadian investment industry [45,72]. The machine learning model deployed in this paper predicted the typical demographic profile of fraud victims as investors who classify as female, have poor financial knowledge, know the advisor from the past and are retired with reasonable accuracy. The logistic regression was the best performing model in predicting fraud victimization for these features with 84% accuracy. The logistic regression classifier made a total of 361 predictions. Out of the 361 predictions, the logistic regression classifier predicted “yes” 52.91% (9.42% + 43.49%) of the time and “no” 47.1% (40.17% + 6.93%) of the time. This is to say that 50.42% of the observation in the sample experienced fraud victimization, while 49.59 were not victimized. More importantly, the model predicted that 9.42% of the victims will be victimized, but the actual results indicated that they were not victimized (i.e., Type 1 error). On the other hand, the model predicted about 6.93% of the victims will not suffer from fraud victimization, when they were actually victimized (i.e., Type 11 error). No other demographics, including age, showed a clear correlation between victimization and financial fraud. Investors who are characterized as having limited financial literacy but a long-time relationship with the advisor had reduced probability of being victimized [4]. While not statistically significant, older adults in general are at greater risk of being victimized [3,4,5,53]. The AUC score of 0.85 illustrates that the logistic regression model did a very good job classifying the positive and negative outcomes at all possible cut off points. Previous researchers profiled typical fraud victim as elderly with increasing reliance on people they are close to (e.g., family members, close friends, someone from their past) to make financial decisions [4]. The thought of running out of money for retirement increases the likelihood of vulnerability and makes them dependent on “experts” for financial advice [67,84].

The scale and magnitude of fraud victimization has led to calls by advocates of fraud victimization and critics alike to streamline Canada’s regulatory systems for more effective regulation of the securities industry [19,49,72,85]. One of the more serious findings is that retirees with poor investment knowledge have an increased risk of being victimized [4]. As previously noted, a myriad of regulators, comprising the provincial securities commission, police agencies and the SROs are responsible for preventing and detecting fraud and enforcing action against criminogenic behavior [86]. Despite the actions taken by these regulators and enforcement agencies, to date there has been no formal action or common front to ensure that the financial ecosystem works efficiently to prevent fraud [86,87]. Canada’s fragmented securities regulatory system has been criticized for ineffective regulation, particularly in the areas of enforcement and investor protection [27,49,86,87,88]. The fragmented nature of Canada’s securities is significant proof that effective self-regulation may be more appropriate than other actions.

Unlike the United States, Canada does not have a national securities regulator like the Securities and Exchange Commission. Where there is an absence of a single set of rules and approaches to compliance, the national SROs could step in and provide leadership to hold their members accountable beyond the statuary minimum. This would be required even if the Cooperative Capital Markets Regulatory System is set up without participation from all the provincial and territorial securities commissions. The proposed unification of the MFDA and IIROC to create new SROs could achieve this outcome by empowering the CSA statutory regulators and restoring public confidence in the market. This paper is written at an opportune time to inform the CSA taskforce about modernizing securities regulation and SRO consultations on investor protection. It is hoped that the findings in this paper will inform the stakeholders on both sides of the regulatory divide to ensure a level playing field for active industry players in the market and to enhance investor protection within Canada’s fragmented regulatory landscape. 

The limitation of this study is that it could have benefitted from a larger dataset. The larger the dataset, the more normally distributed the features would have been. However, the study employed all the cases heard by the IIROC’s hearing panel and coded for the model features. So, while it could be argued that more data could have increased the model power, it is important to note that the model employed the entire population of the data and not a sample of the data. Another possible limitation is that more features could have been added to the model. However, there is the accompanying risk that additional features would have compromised the model’s predictive power. Additional features would have perhaps led to overfitting of the training set and compromise the model accuracy.

In future, researchers should expand on the findings presented in this study to examine the administration of justice for victims of investment fraud. Of particular concern in light of the CSA statutory review is that the SROs are funneling in cases involving criminal elements rather than forwarding them to the Royal Canadian Mounted Police for criminal prosecution. A more qualitative and nuanced approach to this issue would unearth a significant body of knowledge on the administration of justice as it pertains to quasi-criminal offences and their enforcement. The findings could be the basis of a Federal Government project on the administration of justice in Canadian financial regulation. Although a study on the ability of fraud victims to obtain justice is timely, the broader comparative public policy implications concerning investor protection are of more importance.

## Data Availability

Data is available from the authors upon reasonable request.
